# Erythropoietin Ameliorates Rat Experimental Autoimmune Neuritis by Inducing Transforming Growth Factor-Beta in Macrophages

**DOI:** 10.1371/journal.pone.0026280

**Published:** 2011-10-17

**Authors:** Anne K. Mausberg, Gerd Meyer zu Hörste, Thomas Dehmel, Mark Stettner, Helmar C. Lehmann, Kazim A. Sheikh, Bernd C. Kieseier

**Affiliations:** 1 Department of Neurology, Heinrich-Heine-University, Duesseldorf, Germany; 2 Department of Neurology, University of Texas Medical School at Houston, Houston, Texas, United States of America; Julius-Maximilians-Universität Würzburg, Germany

## Abstract

Erythropoietin (EPO) is a pleiotropic cytokine originally identified for its role in erythropoiesis. In addition, in various preclinical models EPO exhibited protective activity against tissue injury. There is an urgent need for potent treatments of autoimmune driven disorders of the peripheral nervous system (PNS), such as the Guillain-Barré syndrome (GBS), a disabling autoimmune disease associated with relevant morbidity and mortality. To test the therapeutic potential of EPO in experimental autoimmune neuritis (EAN) - an animal model of human GBS – immunological and clinical effects were investigated in a preventive and a therapeutic paradigm. Treatment with EPO reduced clinical disease severity and if given therapeutically also shortened the recovery phase of EAN. Clinical findings were mirrored by decreased inflammation within the peripheral nerve, and myelin was well maintained in treated animals. In contrast, EPO increased the number of macrophages especially in later stages of the experimental disease phase. Furthermore, the anti-inflammatory cytokine transforming growth factor (TGF)-beta was upregulated in the treated cohorts. I*n vitro* experiments revealed less proliferation of T cells in the presence of EPO and TGF-beta was moderately induced, while the secretion of other cytokines was almost not altered by EPO. Our data suggest that EPO revealed its beneficial properties by the induction of beneficial macrophages and the modulation of the immune system towards anti-inflammatory responses in the PNS. Further studies are warranted to elaborate the clinical usefulness of EPO for treating immune-mediated neuropathies in affected patients.

## Introduction

Acute inflammatory autoimmune diseases of the peripheral nervous system (PNS) are disabling disorders that - despite advances in treatments of the last decade [1] – are still associated with relevant morbidity and mortality [2]. The most common prototypic acute inflammatory neuropathy is the Guillain–Barré syndrome (GBS) manifesting as a monophasic ascending flaccid tetraparesis with minor sensory deficits [3], [4]. Understanding of the underlying pathomechanisms is still incomplete. There is consensus that GBS results from aberrant cellular and humoral immune responses directed to peripheral nerve antigens resulting in demyelination and/or axonal damage of the peripheral nerve.

Most of our immunopathogenic understanding of GBS was gathered in a well established animal model of this disease, experimental autoimmune neuritis (EAN). The model mimics various clinical and paraclinical features of GBS and is inducible by active immunization with peripheral myelin homogenates, appropriated antigen peptides or transfer of neuritogenic T cells [5]. It offers the possibility to study preclinical effects of novel therapies that may exhibit a clinical benefit in GBS.

Erythropoietin (EPO), known for its role in erythropoiesis, revealed remarkable tissue protective properties in different preclinical models [6]. In experimental autoimmune encephalomyelitis (EAE) treatment with EPO reduces the clinical score, reduces the demyelination and protects from axonal loss [7]. Animals with an ischemic stroke developed smaller infarction zones and reduced inflammation with EPO [8]. Some of these preclinical findings could even be translated into clinical phase II studies, in which peripherally administered EPO exhibited beneficial potential in stroke and patients with MS [9], [10].

Thus, we explored the anti-inflammatory and neuroprotective properties of EPO in autoimmune disorders of the PNS. Therefore, EPO was applied in EAN in a preventive and a therapeutic paradigm and the effect on clinical, histological and immunological measures was assessed.

## Results

### Effect of EPO on the clinical course of experimental autoimmune neuritis (EAN)

**Figure 1:**
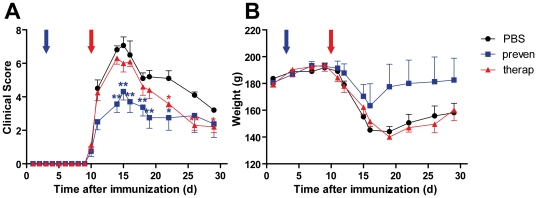
Treatment with EPO ameliorates the clinical course of EAN. EAN was induced in female Lewis rats by subcutaneous immunization with bovine peripheral nerve myelin homogenisates (BPNM, 8 mg/animal) in complete Freud adjuvans. Animals were treated daily with rhEPO (5000 IU/kg) via i.p. injections from day 3 (preven: preventive) or day 10 (therap: therapeutic) after immunization or with vehicle alone (n = 10 per group). Clinical score ranging from 0 (healthy) to 10 (death) was assigned daily in a blinded fashion. On day 15, five randomly selected animals from each group were sacrificed for subsequent histological analysis. Both preventive (red triangles) and therapeutic (blue squares) shortened the duration of severe impairments and disability, while preventive treatment also reduced the maximum EAN severity (A). The preventive treatment also significantly protects from weight loss (B). Clinical score and weight was assessed at the indicated time points (mean ± SEM, * p<0.05; ** p<0.01).

Treatment of EAN with EPO was studied in two different paradigms (Fig. 1). In a preventive paradigm (blue line) EPO was given from day three after immunization. In a therapeutic paradigm (red line) the treatment was started when first clinical symptoms occurred at day 10 post immunisation (p.i). Assessment of the hematocrit at day 29 p.i. revealed that in both treatment paradigms EPO exhibited a similar systemic effect, as indicated by a similar increase in hematocrit values (data not shown).

Compared to PBS injected controls administration of EPO significantly reduced maximal clinical disease severity of EAN in both paradigms (Fig. 1 A). When therapy was initiated at the onset of first clinical symptoms, the duration of the recovery phase was significantly shortened and the remission phase was accelerated. In line with the reduced clinical score during the whole disease course weight loss was less pronounced in the prevention group (Fig. 1B). Thus, EPO ameliorates the clinical signs of autoimmune neuropathy even when therapy was started at later time points, although early treatment has a more prominent effect on disease severity.

### Histological analysis of EPO treatment

**Figure 2:**
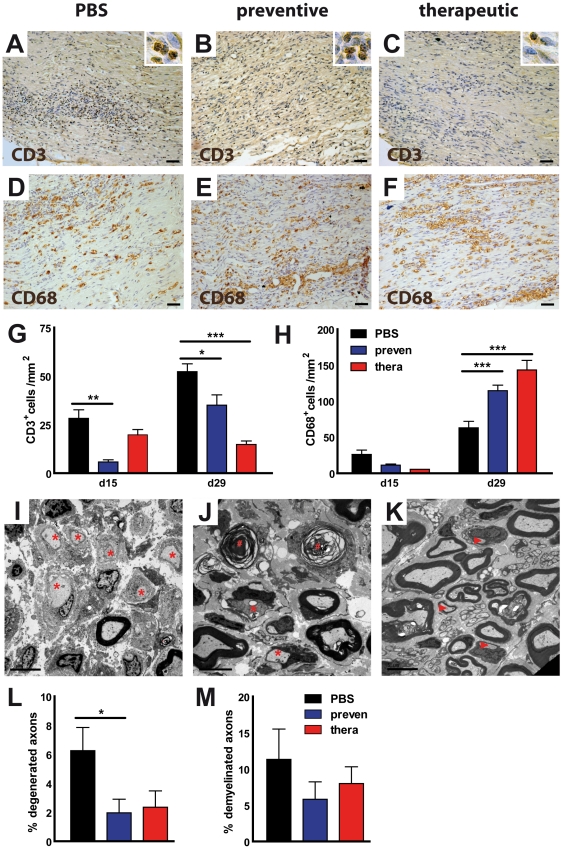
Treatment with EPO improves the peripheral nerve histology. Sections of sciatic nerves obtained at day 29 after immunization were stained for CD3**^+^** T lymphocytes in non-treated (A) and EPO-treated animals (B and C). Scale bar = 50 µm. Immunohistochemistry developed with 3,3′-diaminobenzidine as peroxidase substrate. Insets: Higher magnification of stained T cells showing brown DAB staining in close proximity to blue labelled nuclei. Sections of sciatic nerves obtained at day 29 after immunization were also stained for CD68+ macrophages in non-treated (D) and EPO-treated animals (E and F). Scale bar = 50 µm. Quantitative analysis of d 15 and d 29 revealed the difference in the number of CD3+ T cells (G) and CD68**^+^** macrophages (H). Brachial plexus from control EAN rats (I and J) and EPO treated EAN rats following the therapeutic paradigm (K) were dissected at day 29 and epoxin embedded. Ultrathin sections (80 nm) were stained with toluol blue and analyzed in an electron microscope. Stars: demyelinated axons, number symbols: axon degeneration, arrowheads: remyelinated axons. In control treated EAN rats the nerves have more demyelinated fibres without remyelination (I) and axon degeneration (J). Remyelinated fibres were more frequent in peripheral nerves from EPO treated animals (K). Bars = 5 µm. Quantitative analysis of the percentage of degenerated (L) and demyelinated (M) axons of the plexus nerve in the different groups. preven: preventive; therap: therapeutic. Asterisks indicate significance (mean ± SEM, * p<0.05; *** p<0.001); student's *t* test.

To analyze grade and distribution of endoneural inflammatory infiltrates, sciatic nerve sections were stained for T cells (Fig. 2A–C) and macrophages (Fig. 2D–F). The number of T cells, determined by CD3 staining at day 15 and day 29 was markedly reduced in the treatment group compared to control (Fig. 2G). The preventive treatment showed an approximately 4-fold reduction in T cell numbers at the peak of disease (PBS: 28.2±4.4; prevention: 5.8±1.1), while in the remission phase the effect on T cells was less pronounced (PBS: 52.3±4.1; prevention: 35.1±5.3). The therapeutic administration of EPO reduced the number of T cells at day 15 (19.8±2.7) as well as at day 29 (14.8±1.9). Quantification of T cell staining correlated with the clinical scores in untreated animals compared to the two treatment groups. Infiltration was less pronounced in cohorts with an ameliorated disease. Surprisingly, the CD68 macrophage staining showed disproportionately higher number of macrophages compared to T cells in EPO-treated animals. The CD68 staining at day 29 displayed a massive induction of macrophages in the target organ after preventive and therapeutic treatment (Fig. 2D–F). The striking differences in macrophage number in the treated and untreated groups were not present in the spleen (data not shown). Quantification of macrophages confirmed the significant differences in the EPO-treated groups compared to controls (Fig. 2 H). Although macrophage numbers were not prominent at the peak of disease (PBS: 25.2±6.2; prevention: 10.7±1.5; therapeutic: 4.9±0.1), the remission phase was characterized by a duplication of macrophage numbers under preventive (PBS: 62.7±9.1; prevention: 114.3±7.8) as well as therapeutic conditions (143.3±13.4).

Integrity of myelin was determined in ultrathin sections of the peripheral nerve. Representative electron micrographs depictured pathological changes in EAN nerves. Control nerves without EPO treatment (Fig. 2I,J) showed more demyelinated fibres without remyelination and axon degeneration. Additionally, more interstitial oedema were present. In contrast, in nerves from EPO-treated animals more remyelinated fibres and less demyelination was observable (Fig. 2 K). Pathology, however, exhibited great intraindividual heterogeneity. To address this variability we performed quantitative histological analyses and detected an amelioration of the PNS pathology in EPO-treated animals. The percentage of demyelinated (Fig. 2 M) and hypomyelinated axons (data not shown) showed a non-significant trend towards reduction, while the percentage of degenerating axons was significantly reduced by preventive EPO treatment (Fig. 2 L).

### Proliferation and cytokine profile of EPO treated lymphocytes in vitro

**Figure 3:**
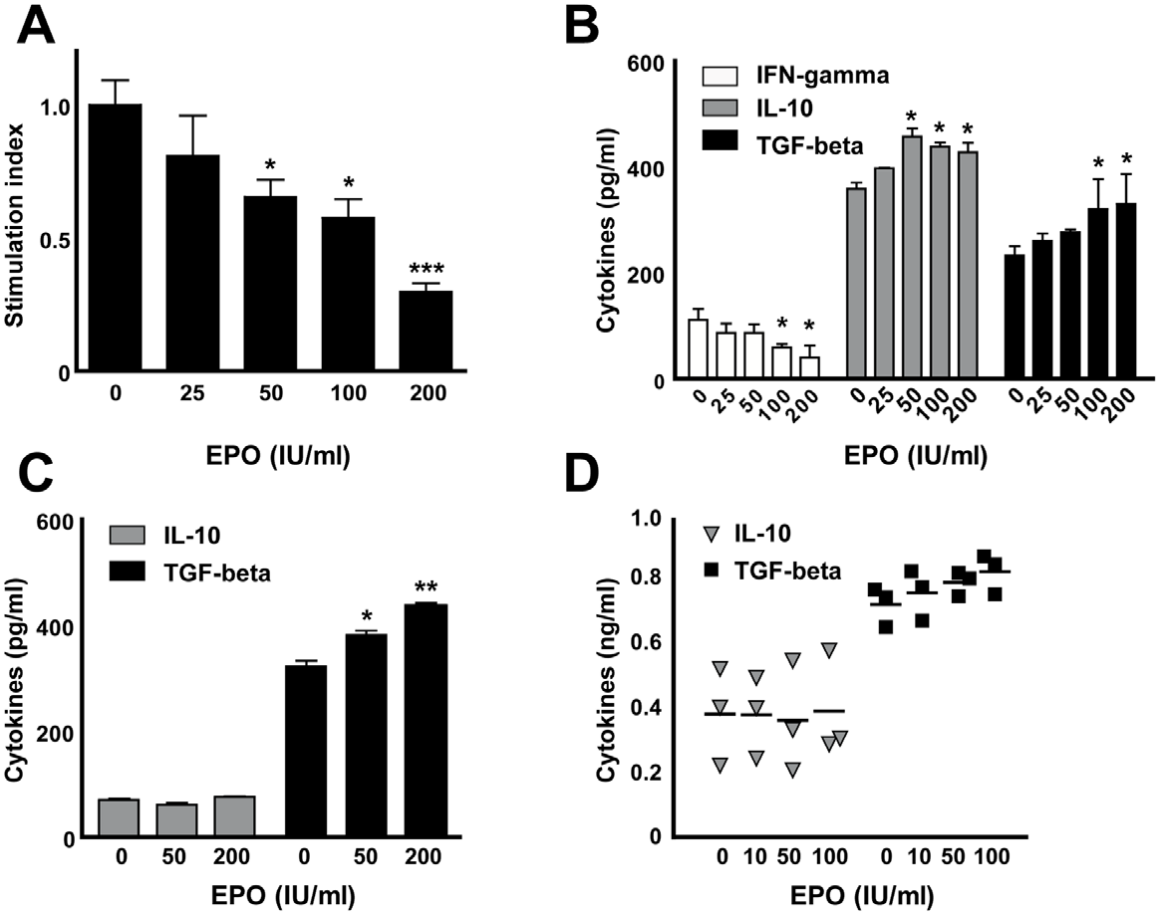
EPO reduces the inflammatory profile of macrophages *in vitro*. Splenocytes from Lewis rats were cultivated with irradiated allogenic Wistar splenocytes and T cell proliferation was measured by ^3^H-thymidine incorporation in the presence of increasing concentrations of EPO (0–200 IU/ml) in quadruplicates. One representative experiment is depicted out of three (A). Supernatants of the proliferation were collected after 72 hours and levels of IFN-gamma, IL-10 and TGF-beta were determined using ELISA (B). Peritoneal macrophages from 2 naïve rats were plated in triplicates in the presence of EPO (0–200 IU/ml) and cytokines were measured via ELISA. Depicted is one representative experiment out of three (C). Peritoneal macrophages from EAN rats at the peak of the disease were cultivated with EPO (0–100 IU/ml) and cytokines were measured. Dots represent mean cytokine levels of single animals determined in duplicates (D). Asterisks indicate significance (mean ± SEM, * p<0.05; ** p<0.01; *** p<0.001); student's *t* test.

The disproportionate numbers of infiltrating T lymphocytes compared to macrophages in the peripheral nerves of EPO-treated animals prompted us to examine the immunomodulatory effects of EPO in leukocyte cultures *in vitro*. Allogenic T cell proliferation of a mixed leukocyte reaction was strongly reduced to approximately 50 percent at higher concentration of EPO (Fig. 3A). In line with this observation, the proinflammatory cytokine IFN-gamma was almost reduced to half in collected cell-supernatants, while the anti-inflammatory cytokines IL-10 and TGF-beta were slightly increased after 72 h of T cell proliferation (Fig. 3B). Comparable results were observed in an antigen specific T cell proliferation and the concomitant supernatant analysis (data not shown). To analyze the effect of EPO on macrophages intraperitoneal cells of naïve rats were cultivated in the presence of EPO and the amount of IL-10 and TGF-beta was assessed (Fig. 3C). In control cultures modest levels of IL-10 were detected but these were not altered by EPO. Altogether the cytokines was expressed in low quantities (below 100 pg/ml). In contrast, a higher TGF-beta level was even present in unstimulated controls (323.3±6.8 pg/ml) and this was further increased by EPO treatment (437.6±2.6 pg/ml at highest concentrations of EPO). To validate our findings in intraperitoneal cells from naïve animals we analyzed peritoneal macrophages from three rats with clinically active EAN at peak of clinical disease independently (Fig. 3D). IL-10 levels were either slightly induced or reduced by EPO in different animals, but in summary not substantially altered. Again, the high levels of TGF-beta were further significantly elevated by EPO stimulation to a comparable amount in all animals. In contrast to T cell proliferation, proliferation of different macrophage subpopulations was not influenced by the presence of EPO, even when cells were prestimulated with IFN-gamma or LPS (data not shown). Taken together, the stimulation with EPO reduces the proliferation as well as inflammatory cytokines *in vitro*, while anti-inflammatory cytokines were induced to a greater extend.

### Detection of TGF-beta in the peripheral inflamed nerve

**Figure 4:**
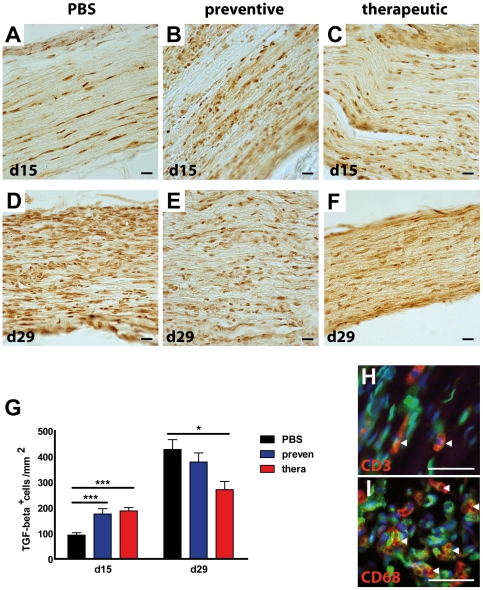
Treatment with EPO induces TGF-beta in the peripheral nerve. Sciatic nerves of untreated and EPO treated animals in a preventive and therapeutic paradigm were dissected at day 15 and day 29 and stained for TGF-beta. While only few cells were positive in the untreated EAN animals (A), preventive (B) and therapeutic (C) treatment with EPO revealed more TGF-beta positive cells in sciatic nerves. TGF-beta staining was even enhanced in the remission phase (D–F). Scale bar = 50 µm TGF beta positive were counted at d 15 and d 29 for quantitative analysis (G). Asterisks indicate significance (mean ± SEM, * p<0.05; *** p<0.001) student's *t* test. preven: preventive; therap: therapeutic. Sciatic nerves dissected at d 29 of therapeutically treated animals were stained for TGF-beta (green) and CD3 (red, H) and CD68 (red, I). White triangles indicate costained cells. Nuclei are visualized using DAPI. Scale bar = 50 µm.

Staining for TGF-beta in sciatic nerves was performed at day 15 and 29 p.i. to assess the possible mechanisms underlying the beneficial effect of EPO (Fig. 4A–F).

At the peak of the clinical disease only a few TGF-beta positive cells were detectable in the sciatic nerve sections of control animals (Fig. 4A,G; PBS: 92.7±9.2). However, even at this early time point the number of TGF-beta positive cells was significantly increased to two folds in EPO-treatment groups (Fig. 4 B,C,G, prevention: 175.3±20.8; therapeutic: 187.1±13.1). In the remission phase at day 29 p.i., numbers of TGF-beta positive cells were increased in all three groups. While in control and preventive regimens a comparable amount of cytokine positive cells were counted at day 29 p.i. (PBS: 427.0±38.2; prevention: 378.2±35.2) the increase in the therapeutic group was not as prominent (270.3±32.1). Costaining of TGF-beta and CD3 (Fig. 4H) or CD68 (Fig. 4I) showed colocalisation of TGF-beta with some CD3**^+^** T lymphocytes. However, most of the TGF-beta staining colocalized with CD68**^+^** cells, indicating that macrophages are the major source of TGF-beta.

## Discussion

Erythroid precursors were believed to be the only cells responsive to EPO, however, an emerging body of evidence reports the EPO receptor (EPOR) to be expressed on a number of different cell types. In contrast to erythrocytes using an EPOR homodimer, in non erythroid tissues the binding of EPO assembles a heteroreceptor complex composed of EPOR and the common beta chain which is also used by other cytokine and growth factor receptors [11]. Hereby, EPO exerts antiapoptotic and proliferative effects on cells of various tissues including the nervous system, kidney, heart and liver (Shaheen and Broxmeyer, 2009). Most interestingly, also immune cells express the EPOR [11], [12]. These findings opened a new avenue of research, specifically under the concept of neuroprotection. The neuroprotective capacity of EPO has been demonstrated in various animal models affecting the nervous system, including stroke (hypoxia/ischemia), spinal cord injury, experimental autoimmune encephalomyelitis (EAE), but also models affecting the peripheral nerve, such as peripheral nerve crush injury [4], [13]–[16]. In the present study we studied the effect in an animal model for immune-mediated PNS diseases - EAN. We demonstrate that EPO reduces clinical disease severity and improves clinical recovery after an immune-mediated hit against the peripheral nerve.

Studies in the central nervous system pointed out that in addition to its neuroprotective properties EPO exhibits immunomodulatory effects. However, it remains elusive at present to which extend EPO exhibits its immunomodulatory capacity on different immune cells. In a recent study in autoimmune model of colitis and the systemic infection with salmonella treatment with EPO concordantly reduced the activation of NF-kappaB and thereby reduced the immune activation [17]. In our study in EAN we demonstrate that EPO induces a reduction of peripheral nerve specific autoimmune reactivity translating into preservation of myelin and axon integrity and reduced cellular infiltration of the PNS. This reduced cellular infiltration of the peripheral nerve was found to be restricted to T cell infiltration, whereas the number of macrophage found within the peripheral nerve was even increased after treatment with EPO. These macrophages were found to express the anti-inflammatory cytokine TGF-beta pointing to a beneficial phenotype of these macrophages, so called M2 macrophages [18].

An obvious question that arises out of this observation is whether EPO induces its effect via mechanisms influencing T cells or via a direct effect on macrophages. Macrophages represent the predominant cell population in the diseased peripheral nerve and can be localized in spinal roots as well as in the more distal segments of nerves. They play a key role as antigen presenting cells and act as effectors destroying myelin [19], [20]. The fundamental need for macrophages in the pathogenesis of the disease - both in the induction and effector stage - was demonstrated by depletion experiments: Macrophage elimination prevented all clinical, electrophysiological, and histological signs of EAN [21], [22]. On the other hand, macrophages are also essential for clearing myelin and axonal debris in the recovery phase of EAN and contribute to the induction of neurotrophic factors [20]. Our data are in line with the findings by Nairz, et al [17]: In their study EPO reduced the production of nitrite oxide (NO) and the pro-inflammatory cytokine TNF-alpha by blocking the transcription factor nuclear factor (NF)-kappaB in thioglycolate induced macrophages. These changes in the macrophage phenotype translated into an impact in clinical disease severity in the colitis model studied, fully in line with our findings in autoimmune neuritis. Clearly, future studies are warranted to dissect the influence of other cell types of the immune system such as B cells and dendritic cells, to evaluate to what extent EPO also determines their immunomodulatory function.

Surprisingly, the number of macrophages within the peripheral nerve increased significantly. This observation is in contrast to the overall reduced infiltration of T cells. It remains elusive at present whether this increase in number is predominantly driven by the recruitment of macrophages from the peripheral immune system or if this represents a dramatic proliferation of endogenous macrophages [23]. However, given the kinetics with highest numbers of macrophages during the recovery phase of the disease an increase in the recruitment of peripheral macrophages appears most likely. Furthermore, the comparable number of macrophages in the spleen with and without EPO at day 15 and 29 and the fact that we did not observe a proliferative effect of EPO on peritoneal macrophages or bone marrow derived macrophages (data not shown) underline that conclusion.

In the present study we could demonstrate that EPO drives macrophages to an anti-inflammatory phenotype. Macrophages produced significantly higher levels of the anti-inflammatory cytokine TGF-beta and we were able to corroborate this observation *in vivo* as well as *in vitro*. Interestingly, these findings could also be validated in peritoneal macrophages derived from EAN animals at the peak of the disease. In the present study, macrophages were obtained from an inflammatory milieu but still comprise the tendency to increase TGF-beta production after EPO stimulation.

TGF-beta is a cytokine modulating immune responses by dampening of the innate immune reaction, but also irreplaceable interfering with the *in vitro* genesis of regulatory T cells (Tregs), another key factor limiting an overreacting immune response. Active TGF-beta is necessary for Tregs to mediate their immunosuppressive properties [24], [25]. Given the importance in mediating peripheral tolerance it is consequential that blocking of TGF-beta worsens the clinical outcome in various models for autoimmunity like experimental autoimmune encephalitis, collagen induced arthritis and the non obese diabetic mice (NOD) [24], [26]–[28].

Macrophages as well as T cells are capable of producing TGF-beta [29], [30], as it has also been demonstrated in the context of EAN before [31]. In our study we could localize anti-TGF-beta staining to CD3 as well as CD68 positive cells, however, overall macrophages were identified as the main source of this anti-inflammatory cytokine within the inflamed peripheral nerve.

While EPO obviously had a significant impact on TGF-beta expression within the peripheral nerve, our *in vitro* studies revealed only a small increase in the overall amount of TGF-beta production by macrophages when measured by ELISA. A possible explanation for this discrepancy could be the short range of operation for cytokines like TGF-beta. *In vitro* experiments do not account for the local effects that cytokines have in the target organ. Even a small amount of cytokine locally may be sufficient to protect the nerve from massive inflammatory infiltration.

Taken together it is most likely that the local increase of TGF-beta represents an anti-inflammatory milieu protecting the PNS of EPO treated animals; it correlates with the beneficial outcome considering clinical course and the preserved myelin integrity. Our data do not support any concept of a direct neuroprotective effect of EPO in EAN, however, our model system is clearly inflammatory driven and inadequate to be used as a model to study neurodegeneration. Future studies are clearly warranted to unravel the potential of EPO as an interesting candidate in treating autoimmune diseases of the PNS.

## Materials and Methods

### Induction of experimental autoimmune neuritis

Animal experimentation was approved by local state authorities (Landesamt fuer Natur, Umwelt und Verbraucherschutz Nordrhein-Westfalen) under the approvalreference number 84-02.04.2011.A112. EAN was induced as previously described [32]. Briefly, female Lewis rats (8 weeks, Charles River Laboratories) received subcutaneous injections (200 µl) at the back of 6 mg of bovine peripheral nerve myelin (BPNM) generated as previously described [33] in 100 µl PBS emulsified with 100 µl complete Freund's adjuvant (CFA, Difco) containing 1 mg/ml heat inactivated Mycobacterium tuberculosis (H37Ra). A modified EAN score [34] was applied: 0 no impairments, 1 reduced tone of the tail, 2 limp tail, 3 absent righting reflex, 4 gait ataxia, 5 mild paraparesis, 6 moderate paraparesis, 7 severe paraparesis or paraplegia, 8 tetraparesis, 9 moribund, 10 death due to neuropathy.

### EPO treatment

EPO (Epoetin Alfa, Ratiopharm, Germany) in 2,000 U/ml vial stock was used for treatment. Administration of EPO was either starting at day 3 after immunization (preventive) or starting at day 10 after immunization (therapeutic). Animals were treated daily with intraperitoneal (i.p.) EPO at a dose of 5000 IU/kg/day, as established before [7] or as controls with an equal volume of PBS.

### Histology

At peak of clinical disease activity, day 15 post immunization (p.i.), and at day 29 p.i. a randomly chosen half of all experimental groups of animals was sacrificed and perfused with PBS followed by 4% paraformaldehyd. Spleens and sciatic nerves were dissected, post-fixed with paraformaldehyd overnight and paraffin embedded. 10 µm sections (standard microtome HM355S, Microm, Walldorf, Germany) were costained with haematoxylin/eosin (HE) and rabbit anti-CD3 antibody (DAKO, Glostrup, Denmark) or mouse anti-CD68 antibody (Serotec, Duesseldorf, Germany) using matching biotinylated secondary antibodies (Vector, Peterborough, UK) followed by an avidin-biotin-horseradish peroxidise complex (DAB Kit, DAKO) using 3,3′-diaminobenzidine (DAB) as peroxidise substrate according to manufacturer's instructions. Transforming growth factor (TGF)-beta staining (rabbit anti-TGF-beta antibody, Santa Cruz, Heidelberg, Germany) was performed without HE staining. For fluorescent staining FITC and Alexa Flour 633 conjugated secondary antibodies were applied (Invitrogen, Darmstadt, Germany) and slices were covered using Vectashield (Vector) mounting medium with or without 4,6′diamidino-2-phenylindole (DAPI). For quantitative analysis of positive cells infiltrating the nerve three entire nerve longitudinal sections from each animal were photographed with a high magnification (Axioplan 2, Zeiss), the area covered by the tissue was determined and the number of positive cells per mm^2^ was counted using ImageJ software (v1.44, NIH).

For morphological studies and analysis of axonal degeneration the brachial plexus was dissected from perfused animals (n = 5 per group), post-fixed in 4% paraformaldehyd over night and embedded in epoxy resin, as previously described [35]. Toluidine blue stained semi-thin (1 µm) and ultrathin (200 nm) sections were examined by light and electron microscopy, respectively. For statistical analysis of axonal pathology, semi-thin sections of plexus nerves were photographed and the photographs were photomerged using Photoshop CS3 (Adobe). The total number of normally myelinated, hypomyelinated, fully demyelinated and degenerating axons was manually counted by an investigator blinded towards previous treatment using the CellCounter plugin of ImageJ. Each individual axon was manually marked and automatically counted. Physiologically unmyelinated axons (diameter <1 µm) and Remak-bundle fibres were not included. Degenerating axons were morphologically defined as axonal remnants with partially intact Schwann cell ensheathment, but with lost axonal interior structure. The percentage of intact and abnormal axons was calculated and compared between groups.

### T cell proliferation assay

Spleens of rats were dissected under sterile conditions and passed through a 40 µm cell strainer followed by ammonium chloride based erythrocyte lysis (BD Bioscience, Heidelberg, Germany). Derived splenocytes were cultured in flat bottom 96-well plates in standard T cell medium (IMDM with 5% FCS, 2 mM L-glutamine and 50 µM 2-ME, Invitrogen). Responder cells from Lewis rats (1×10^5^/well) were cocultured with irradiated (1000 rad) allogenic splenocytes of Wistar rats as stimulator cells (1×10^5^/well). EPO was added during the culture period with increasing concentration from 0.3 to 200 IU/ml. For antigen specific T cell proliferation spleens of EAN rats were dissected at day 15 p.i. under sterile conditions cultivated as described above in the presence of BPNM (10 µg/ml). T cell proliferation was measured via [^3^H] thymidine incorporation during the last 24 h of a four day incubation. Liquid scintillation counting (BetaPlate1205, Perkin Elmer, Rodgau, Germany) given as counts per minute (cpm) of quadruplicate test cultures ± SEM was measured. Stimulation index was calculated as ratio of the cpm at the indicated EPO concentrations to the proliferation of cells in the absence of EPO.

### Macrophage culture

Peritoneal macrophages were prepared as described previously [36]. Briefly, 2–3 month old Lewis rats were injected i.p. with ice-cold PBS and fluids were recollected. Macrophages were isolated from untreated rats as well as EAN rats at day 15. Obtained cells (2×10^4^) were cultivated in 96-well plates for 48 hours with DMEM (10% FCS, 2 mM L-glutamine, 100 U/ml Penicillin und 100 µg/ml Streptomycin) with increasing concentrations of EPO (0–200 IU/ml).

### Cytokine quantification

Supernatants were collected from T cell proliferation assays after 72 hours or from macrophage cultures after 48 hours. Interleukin (IL)-10 and interferon (IFN)-gamma enzyme linked immunosorbant assays (ELISA) were performed due to the manufacturer's protocol (BD Bioscience). TGF-beta ELISA was derived from R&D Systems (Wiesbaden, Germany). In all analyzed samples latent TGF-beta was activated according to the manufacturers' protocol. Supernatants as well as standard curve were measured in duplicates on a Rainbow Photometer (Tecan, Crailsheim, Germany) using easyWIN software. Concentrations are given as mean ± SEM.

### Data analysis

Data were statistically analyzed using GraphPadPrism 5.0 (GraphPad Software). The Wilcoxon-Mann-Whitney was used to test for statistically significant differences in clinical score values. Student's t-test for unrelated samples was used to test for statistically significant differences in all other analyses. Differences were considered significant at p-values<0.05.
